# Domestic Correspondence

**Published:** 1884-05

**Authors:** 


### Domestic Correspondence.

NEW YORK, MARCH 19, 1884.

After a winter exceptionally eventful and exciting in many respects, the prospect of a tranquil spring in the medical world is peculiarly grateful to every member of our professional community. We have all had enough, and more than enough, of medical politics. Allusions to the Code are beginning to be so strictly tabooed that "codophobia" may be said to be epidemic. To be sure, party lines are still drawn rather severely; we have our County Medical "Association" as well as "Society," each determined to "fight it out" on their individual ethical line for an indefinite number of summers. But party spirit is no longer at fever heat. Good-nature prevails everywhere. Fierce denunciation and frantic cavillings have, for the present at least, most happily drifted into some little semblance of brotherly love. A few paltry ebullitions of spleen have abortively spluttered up from various quarters at long intervals, but have been greeted either with manifest disfavor or thorough lack of interest. As a genial old practitioner remarked to me the other day: "Exit, the Kilkenny cats! Enter, the lion and the lamb!" Now, all this may be the lull before the storm, but everybody is nevertheless heartily thankful for the lull.

The Druggists' Protective Union, however, has succeeded in raising quite a respectable tempest in the pharmaceutical teapot by an organized and plainly avowed effort to boycott certain members of their guild. The gist of the Union's "grievance" is to be found in the sale of patent medicines. The custom of selling them below the published rates seemed to be growing. The purpose of the "Union" is to pledge the proprietors of these nostrums to refuse to deliver any goods to those who persist in selling at a discount. The actual result accomplished has been a free advertisement for several old and well established firms, which are thoroughly equipped for such a fight. The scope of the contest has been extended to include all drugs; and it is certainly astonishing how many of our wholesale dealers have been drawn into a movement to impose a universal arbitrary scale of prices upon the retail trade. The incident scarcely requires comment, as nothing will be secured but the usual ruin of various small fry. That such a combination has assumed such proportions, does not merely show the overcrowded state of the drug trade in New York. It affords the most pitiable proof that legitimate pharmacy has not only recognized, but practically taken a second place to barefaced quackery.

The management of the Skin and Cancer Hospital (which has lately issued its first annual report, exhibiting a very respectable amount of honest work) has judiciously decided to restrict the benefits of the institution to cancer cases alone. It is designed to erect in the near future suitable suburban pavilions, as the present cramped quarters on East Thirty-fourth street have proved wholly inadequate. A "kirmess" (æsthetic for "fair") will shortly be given at Delmonico's for this purpose. A similar affair last year was very successful. An amateur dramatic entertainment will also be given this week, at the Academy of Music, in aid of an hospital for chronic invalids. Our chronic incurables seem at last to have touched the public conscience, and the profession must see to it that all the energy is utilized. Opportunities for exact, careful, continuous, scientific observation of chronic maladies are painfully few in proportion to their importance. The tendency of medical thought toward the collective investigation of disease will perhaps furnish a new impetus in this direction.

The County Medical Society have been quick to take the hint of their President, Dr. Vanderpoel, in his inaugural address, on this very subject. The following questions have been submitted to each member of the society, and discussion on the result of the inquiry is the special order for the stated meeting on May 24:

1. How many cases of intestinal obstruction have come under your observation, and what particular symptoms were manifested in each case?

2. Have you performed an operation for the relief of intestinal obstruction due to peritoneal adhesions, and if so, what was the result in each case?

3. Have you treated any cases of intestinal obstruction, partial or complete, where the so-called pathognomonic symptoms, such as stercoraceous vomiting and obstinate constipation, were absent?

At a recent meeting of the Academy of Medicine, Dr. G. B. Fowler gave an interesting *résumé* and demonstration of the various methods of detecting albumen in the urine. He called attention to the point, that while heat will detect two-tenths per cent. of albumen, the urine should be nearly neutral, as an excess of acid or alkali will convert albumen into a form which is soluble in heat. Nitric acid, picric acid, and sodium tungstate will each detect one-tenth per cent. Picric acid, however, not only stains the fingers, but alters the color of the urine. He discarded Roberts' acidulated brine. (This last, by the way, has been very popular in many of our hospitals). Potassio-mercuric iodide was by all odds the most delicate, detecting one-hundredth per cent. of albumen. All of the recent tests give a reaction with peptones and quinine, to obviate which he suggested a simple test for peptonuria, and an inquiry as to whether the drug had been lately taken by the patient. The slightest trace of albumen deserved watching. We should not rely on a single test. As the significance of a trace of albumen in the urine was not understood, the value of these delicate tests could not be settled at present.

Dr. C. A. Doremus suggested meta-phosphoric acid as perhaps a more delicate test than any yet advanced. The general opinion of the members seemed to favor the careful use of the old heat and nitric acid tests.

